# Home‐based chemotherapy for stage III colon cancer patients in Thailand: Cost‐utility and budget impact analyses

**DOI:** 10.1002/cam4.3690

**Published:** 2020-12-30

**Authors:** Nattanichcha Kulthanachairojana, Phichai Chansriwong, Nintita Sripaiboonkij Thokanit, Suwannee Sirilerttrakul, Nopakan Wannakansophon, Suthira Taychakhoonavudh

**Affiliations:** ^1^ Department of Social and Administrative Pharmacy Faculty of Pharmaceutical Sciences Chulalongkorn University Bangkok Thailand; ^2^ Medical Oncology Unit Department of Medicine Faculty of Medicine Ramathibodi Hospital Mahidol University Bangkok Thailand; ^3^ Ramathibodi Comprehensive Cancer Center Ramathibodi Hospital Mahidol University Bangkok Thailand; ^4^ Department of Nursing Ramathibodi Hospital Mahidol University Bangkok Thailand

**Keywords:** colon cancer, cost‐utility analysis, home‐based chemotherapy, portable infusion pump

## Abstract

Home‐based chemotherapy (HC) is a new treatment alternative to hospital‐based chemotherapy treatment (IP) and is administered via portable intravenous pumps at the patient's home. HC reduces the demand for inpatient bed capacity in hospitals and reduces the cost of an infusion. This study takes a societal perspective while conducting the cost‐utility and budget impact analyses (BIA) of HC and IP with an mFOLFOX6 regimen on patients with stage III colon cancer. We conducted a cost‐utility analysis with a 6‐month time horizon. The parameter inputs for the model were gathered from a retrospective cohort study on patients diagnosed with stage III colon cancer at Ramathibodi Hospital, Bangkok. The resource usage of HC and IP was determined based on medical records. The per‐unit direct medical, home health service, and adverse events (AE) management costs were gathered from the standard cost list. The health outcome of treatment was measured in terms of quality‐adjusted life years. Disutility related to AE was calculated. We conducted a sensitivity analysis for the uncertainty results and performed BIA based on the societal perspective on a 1‐year time horizon. HC provided a cost‐saving of $1,513.37 per patient for the period of treatment. Thus, assuming 526 patients per year, the use of HC could achieve a cumulative annual cost‐saving of $828,436. HC is a cost‐saving strategy compared to IP for stage III colon cancer treatment. We recommend that the service reimbursement should include national standardization in chemotherapy regimens as well as practice guidelines and protocols to prevent serious AEs.

## Graphical abstract

Home‐based chemotherapy is a cost‐saving strategy compared to hospital‐based chemotherapy for stage III colon cancer treatment. We recommend that the service reimbursement should include national standardization in chemotherapy regimens as well as practice guidelines and protocols to prevent serious adverse events.

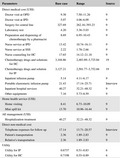

## 1 INTRODUCTION

Resource scarcity is a long‐standing issue in the healthcare sector. Given the high cost of health expenditures, searching for innovative ideas to provide healthcare services more efficiently is common. With an increasing number of patients being diagnosed with cancer, the hospitals in Thailand are working at their full capacity to provide chemotherapy services to them. However, long wait times for the next chemotherapy treatment cycle are common and unquestionably affect patients' treatment outcome and satisfaction.^1^, ^2^

For stage III colon cancer patients, 5‐fluorouracil (5‐FU)–based adjuvant chemotherapy is a typical type of treatments similar to the mFOLFOX6 regimen. It is a combination between 5‐FU, leucovorin, and oxaliplatin. Usually, patients need to be hospitalized due to the continuous infusion in medication over 46 h. Frequent hospitalization is required since the treatment is scheduled for every 2 weeks.^3^, ^4^

In Thailand, the use of HC began in 2016 at Ramathibodi hospital, which was the first healthcare facility in the country to offer HC to patients. HC was developed in order to solve the problem of resource scarcity resulting from frequent hospitalizations of patients receiving the mFOLFOX6 regimen. The services progress from IP practice since 5‐FU is available in a portable pump connected to a central venous access port (CV port) that can perform the ongoing infusion for patients at their home. When the medication is complete, the pump can be removed via an outpatient visit.^5^, ^6^ Thus, for patients receiving the mFOLFOX6 regimen, there is no need for hospitalization. The previous study showed that the implementation of HC has substantially improved patients' quality of life and their satisfaction. In addition, the cost of chemotherapy treatment can be reduced with HC.^6^

Other evidence has shown that HC is safe and offers potential benefits in both clinical and humanistic aspects compared to traditional chemotherapy administrated in hospital settings.^6^, ^7^ However, an analysis of the economic and financial aspects of HC is still lacking. The lack of evidence affects the decisions regarding an HC reimbursement policy in Thailand. Thus, the aim of this study is to perform an economic evaluation and a budget impact analysis (BIA) on home‐based and hospital‐ or inpatient‐based chemotherapy (IP) administration in stage III colon cancer patients in Thailand. The results can be used to inform the decisions about HC reimbursement policy in Thailand with regard to increased patient access to HC treatment.

## 2 METHOD

This economic evaluation study takes a societal perspective and conducts a cost‐utility analysis, comparing the administration of a mFOLFOX6 regimen among patients with stage III colon cancer in HC and IP treatment settings. Costs and health outcomes were gathered from a retrospective cohort study on patients diagnosed with stage III colon cancer at Ramathibodi Hospital. The patients were treated with mFOLFOX6 for 12 cycles (6 months) following the guidelines of the National Comprehensive Cancer Network (NCCN).^8^ Quality‐adjusted life years (QALYs) were calculated from the utility values of each health condition, including a patient's adverse events (AE) associated with the intervention. The analysis was done in Microsoft Excel 2013 and STATA version 13.

### 2.1 Patients and intervention

In this study, patients in the HC group underwent the protocol specified in the Home Chemotherapy RAMA Model (HCRM) and patients in the IP group received chemotherapy in standard inpatient settings.^4^ The HCRM excluded patients with an Eastern Cooperative Oncology Group performance score of more than one. Patients were excluded if they lived further than 1 h drive from the hospital, or were not able to monitor their treatment and the HC equipment, according to the protocol. However, the first cycle of chemotherapy was administered to both groups in inpatient settings. Patients were scheduled to receive 12 cycles of a mFOLFOX6 regimen every 2 weeks, for 6 months, as specified in the cancer treatment protocol of the NCCN and National Health Security Office (NHSO) in Thailand.^4^, ^8^

The study was a retrospective cohort study conducted on patients diagnosed with stage III colon cancer. Fifty‐three patients with HC and 264 patients with IP were included in the study. They were selected by matching the propensity score to age, gender, and comorbidity (anemia, cardiovascular disease, chronic hepatic syndrome, hepatitis, diabetes, gout, hyperlipemia, hypertension, mental disorder, and malnutrition conditions), matching one to many without replacement. For HC, a multidisciplinary team led by an oncologist and an oncology nurse, assessed the patient's health status. The surgical procedure of central venous (CV) port implanting was performed. For chemotherapy administration, the HC patients received their chemotherapy at the short stay service (SSS) for the oxaliplatin, leucovorin, and 5‐FU medication, before they were sent home with a 5‐FU portable elastomeric infusion pump. Patients were advised to visit the SSS or any healthcare facility to remove the pump when its purpose was served. Patients in the IP group were not implanted with the CV port. Instead, the drugs were administered through peripheral infusion throughout the treatment cycle.

### 2.2 Costs

Our analysis adopts a societal perspective and considers both direct medical and direct non‐medical costs. The direct medical costs include costs of healthcare personnel, laboratory tests, drugs and drug administrations, nursing time, surgical procedure for central line, dispensing fees, home health services, AE management, and equipment. The direct non‐medical costs include telephone expenses and costs of transportation. Data on resource utilization for both the groups were primarily collected from a retrospective cohort study of HC and IP patients at Ramathibodi Hospital. Resource unitization was averaged for each patient before being input in the model to reflect the average resource usage of HC and IP groups. Cost was estimated based on the number of patient visits following the HCRM process of HC and IP treatment and the standard cost list of the Health Intervention and Technology Assessment Program.^9^ Cost of drugs were retrieved from the Drug and Medical Supply Information Center (DMSIC) website,^10^ while the cost of the portable elastomeric infusion pump was estimated based on the market price, since both HC and IP patients received the chemotherapy drug for the 6‐month period. Costs of the chemotherapy drug were calculated. All costs are in US dollars and were adjusted using the Consumer Price Index for the year 2019. Costs of nurse counseling for HC that were not included in the standard cost list were estimated based on time‐driven activity‐based costing (TDABC). Namely, TDABC was adopted for cost estimation and quantified into a monetary value. The cost parameter is presented in Table 1.

**TABLE 1:** Model parameters.

Parameters	Base case	Range	Source
Direct medical cost (US$)			
Doctor visit at OPD	9.38	7.50–11.26	9
Doctor visit at IPD	5.07	4.06–6.09	9
Surgery for central line	327.69	262.16–393.23	9
Laboratory test	4.20	3.36–5.03	9
Preparation and dispensing of chemotherapy by a pharmacist	8.69	6.95–10.43	9
Nurse service at IPD	13.42	10.74–16.11	9
Nurse service at SSS	2.22	1.78–2.66	9
Nurse counseling for HC	17.65	14.12–21.18	9
Chemotherapy drugs and solutions for HC	3,104.86	2,483.89–3,725.84	19
Chemotherapy drugs and solution for IP	3,127.21	2,501.77–3,752.66	19
Inpatient infusion pump	5.14	4.11–6.17	9
Portable elastomeric infusion pump	21.43	17.14–25.71	Interview
Inpatient hospital services	40.27	32.21–48.32	9
Other equipments	7.16	5.73–8.59	9
Home health service (US$)			
Home visiting	8.41	6.73–10.09	9
Mini spill kit	13.70	10.96–16.44	9
AE management (US$)			
Hospitalization treatment	40.27	32.21–48.32	9
Direct non‐medical cost			
Telephone expenses for follow up	17.14	13.71–20.57	Interview
Patient's transportation	2.36	1.89–2.83	9
Relative's transportation	2.36	1.89–2.83	9
Utility			
Utility for IP	0.6737	0.51–0.83	6
Utility for HC	0.7198	0.55–0.89	6

Abbreviations: HC, home‐based chemotherapy; IP, hospital‐based chemotherapy; IPD, inpatient visit; OPD, outpatient visit; SSS, short stay service.

### 2.3 Health outcomes

QALYs was used as the measure of health outcomes and was calculated from the utility score (quality‐of‐life weight) multiplied by a 6‐month time horizon (12‐cycle cancer treatment). Utility scores were calculated based on the Functional Assessment of Cancer Therapy‐Colorectal score and these data were collected from patients diagnosed with colorectal cancer at the Ramathibodi Hospital.^6^, ^11^ AE rates associated with HC and IP were collected from the electronic medical records of HC and IP patients. Utility decrement for AE was incorporated into the analysis for any such case. The utility decrements adopted in this model have been described in detail in previous studies (Table 2).^12^, ^13^

**TABLE 2:** % of AEs and hospitalization day of HC and IP

Events	Hospitalization days (range)	Utility reduction	AEs (%) (range)	
			HC (n = 53)	IP (n = 264)
Sepsis	14.56 (5–44)	−50%	0.00	2.27 (0–2.27)
Thrombocytopenia	1.00	−45%	0.00	0.76 (0–0.95)
Agranulocytosis	12.33 (1–32)	−45%	0.00	1.14 (0–1.14)
Pulmonary embolism	4.30 (1–44)	−50%	0.00	1.14 (0–1.14)
Phlebitis	4.35 (1–44)	−50%	0.00	3.79 (1.89–3.81)
Vein thromboembolism	2.09 (1–9)	−50%	0.00	0.76 (0–1.03)
Pneumonia	12.75 (4–35)	−50%	0.00	0.76 (0–0.95)
Nausea and vomiting	6.00 (1–6)	−50%	0.00	0.38 (0–0.38)
Fever	3.18 (1–6)	−50%	3.77 (2.86–4.12)	4.17 (3.48–5.71)
Vascular complication	2.00 (1–2)	−50%	0.00	0.38 (0–0.95)
Anaphylacsis	3.00 (2–4)	−50%	0.00	0.76 (0–0.95)
Antineoplastic AEs	2.86 (2–5)	−50%	0.00	1.89 (0.95–3.09)
Anemia	3.56 (1–23)	−45%	0.00 (0–2.86)	4.17 (1.89–5.70)

Abbreviations: AEs, adverse events; HC, home‐based chemotherapy; IP, hospital‐based chemotherapy.

### 2.4 Economic analysis

For each patient's treatment, the patient's costs and outcomes during the 6‐month time horizon were calculated. Incremental cost‐effectiveness ratios (ICERs) of cost per QALY gained were also calculated.

### 2.5 Sensitivity analysis

We conducted deterministic sensitivity analyses for robustness, in which each value of the model parameters was varied to investigate the impact. We also conducted scenario analyses by changing the number of chemotherapy cycles to six, as not all patients in the full, real‐world population complete the 12 cycles of chemotherapy. Moreover, we examined the potential effect of the change when the first cycle of chemotherapy was administered at home, instead of at the hospital for the HC group.

To investigate the overall robustness of the input values, we employed a probabilistic sensitivity analysis (PSA) in which one thousand Monte Carlo simulations were executed. For the PSA, each simulation parameter was randomly selected from the model inputs distribution, with beta distributions for the probabilities in clinical and health utilities, and gamma distributions for costs. The values and ranges of the model parameters are shown in Tables 1 and 2.

### 2.6 Budget impact analysis (BIA)

To assess the financial sustainability of the HC service, BIA was used to compare direct medical, direct non‐medical, and indirect costs of the two scenarios: one that implemented HC and one without the HC alternative. The analysis was performed considering a 1‐year time horizon. The size of the target population was defined based on the incidence of metastasis colorectal cancer in Thailand from the study by Pattanaphesaj and Teerawattananon.^14^ The resource utilizations and cost data used in the cost‐utility analysis were also used in the budget impact model. Patient's and relative's opportunity costs were estimated based on the gross domestic product (GDP) per capita ($4,571.43). We assumed that patients with HC would still be able to work at half their productivity level during HC. We conducted a univariate sensitivity analysis to test the robustness of the results. The percentage of patients that shifted to HC was varied to investigate 20%, 50%, and 100% shifts.

## 3 RESULTS

### 3.1 Costs, QALYs, and cost‐utility analysis

The demographic characteristics of the patients undergoing HC or IP are presented in Table 3. The average age was 58.84 ± 11.07 and 61.15 ± 10.85 years for HC and IP, respectively. The majority of HC patients were females (50.94%). While the majority of IP patient were males (52.27%). Hypertension was the highest comorbidity in both groups. All patients received 12 cycles of chemotherapy for 6 months. The costs, QALYs, and ICER results of HC and IP chemotherapy administration are listed in Table 4. The total cost of HC was lower than that of IP ($4,493.59 and $6,006.96, respectively). HC was also linked to better QALYs compared to IP (0.3568 and 0.3362, respectively). Thus, HC emerges as the dominant strategy with an estimated cost‐saving of $1,513.37 per patient per period of treatment. The direct medical costs of inpatient treatment ($1,328.86 savings) were attributed as the largest cost reduction component, followed by the costs of nurse services ($400.87 savings) (Table 5). Patients in the IP setting incurred 17.5% higher costs in transportation to hospitals compared to those who availed HC treatment. Since, the patients should go to reserve the bed at hospital before IP treatment.

**TABLE 3:** Demographic characteristics

Demographic	HC (n = 53)	IP (n = 264)
Age (mean ± SD)	58.84 ± 11.07	61.15 ± 10.85
Gender (female) (%)	27 (50.94)	126 (47.73)
Comorbidity (%)		
Anemia	3 (5.66)	25 (9.47)
Cardiovascular disease	1 (1.89)	7 (2.65)
Chronic hepatic syndrome	1 (1.89)	8 (3.03)
Hepatitis	3 (5.66)	18 (6.82)
Diabetes	9 (16.98)	54 (20.45)
Gout	2 (3.77)	12 (4.55)
Hyperlipemia	6 (11.32)	20 (7.58)
Hypertension	23 (43.40)	129 (48.86)
Mental disorder	2 (3.77)	11 (4.17)
Malnutrition conditions	5 (9.43)	33 (12.50)

Abbreviations: HC, home‐based chemotherapy; IP, hospital‐based chemotherapy.

**TABLE 4:** Baseline results

Alternatives	Costs (US$)	QALYs	ICER
Hospital‐based chemotherapy	6,006.96	0.3362	‐
Home‐based chemotherapy	4,493.59	0.3598	Dominant

Abbreviations: ICER, incremental cost‐effectiveness ratio; QALY, quality‐adjusted life‐year.

**TABLE 5:** Cost categories

Categories	IP costs (US$)	HC costs (US$)	Differences (US$)
Laboratory tests	50.34	50.34	‐
Drugs and solutions	3,127.21	3,104.86	−22.35
Pharmacy services	208.54	104.27	−104.27
Doctor services	173.40	117.62	−55.78
Nurse services	483.22	82.35	−400.87
Inpatient services	1,449.67	120.81	−1,328.86
Patient transportation	113.40	115.76	2.36
Relative transportation	170.09	118.12	−51.97
Chemotherapy administration equipment	185.14	577.11	391.97
Home visits	‐	1.18	1.18
AE management	28.79	5.28	−23.52
Other costs	17.14	95.89	78.75
Total	6,006.96	4,493.59	−1,513.37

Abbreviations: HC, home‐based chemotherapy; IP, hospital‐based chemotherapy.

### 3.2 Sensitivity analyses

The one‐way sensitivity analyses results show that the parameters with the strongest influence on ICERs were AE in IP. Other significant parameters that impacted the ICER results were number of hospitalization days resulting from AE, medical costs, costs of equipment and costs of transportation. Overall, HC was the dominant strategy in all the sensitivity analyses that were conducted. Furthermore, from the 1,000 Monte Carlo simulations conducted, 827 iterations resulted in HC as the dominant strategy. The scenario analyses, including the scenario of six cycles of treatment instead of 12 cycles of treatment, as well as the case when all cycles in the HC group were performed at home, also show that HC is the dominant strategy (Table 6).

**TABLE 6:** Scenario analysis results

Scenarios	Alternatives	Costs (US$)	QALYs	ICER
Base case	IP	6,006.96	0.3362	‐
	HC	4,493.59	0.3598	Dominant
HC were administered in home for all cycles	IP	6,006.96	0.3362	‐
	HC	4,359.77	0.3598	Dominant
6‐cycle of treatment	IP	3,055.91	0.1678	‐
	HC	2,368.37	0.1798	Dominant

Abbreviations: HC, home‐based chemotherapy; ICER: incremental cost‐effectiveness ratio; IP, hospital‐based chemotherapy; QALY, quality‐adjusted life‐year.

### 3.3 Budget impact analysis (BIA)

The BIA showed that the use of the HC treatment leads to an annual cost‐saving in both direct and indirect costs. If all patients undergoing a mFOLFOX6 regimen were shifted to HC, the total cost savings would be $828,436 per year. The reduction in costs is mainly due to less hospitalizations for chemotherapy infusion, as well as less AEs. Using HC would also save $32,918 per year in indirect costs, which result from the fact that patients who receive HC could remain in the workforce. However, if only 50% and 20% of the patients undergoing a mFOLFOX6 regimen were to be shifted to HC, the savings would be lower ($414,218 and $165,687 savings per year, respectively).

## 4 DISCUSSION

This is, to date, the first long‐term economic evaluation study comparing the administration of chemotherapy at home with the standard inpatient treatment in Thailand. It is found that HC is the dominant strategy that provides better health outcomes for patients at a lower cost of treatment, when compared to IP, which is the standard practice in Thailand. The results are confirmed to be robust in both deterministic and probabilistic sensitivity analyses—82% of the simulations showed that HC remains a cost‐saving option, even when input parameters are adjusted.

The results from our study are consistent with those from previous studies, which show that HC is a cost‐saving alternative compared to IP. A South Korean study by Joo et al. showed that the total cost of HC is 16% lower than that of IP; while HC involved higher transportation costs, the total costs were offset by the lower total medical costs required for HC.^15^ A study in Italy found that HC had lower costs than IP in terms of medical examination, nursing, and food; while the total cost of HC in this study was higher, this was explained by the higher price of the electronic pump ($420 per cycle of treatment), which was used instead of the elastomeric pump used in Thailand.

In confirming HC's effectiveness in reducing costs, this study extends the treatment period used in the analysis of cost and outcomes, from a single chemotherapy session, as done in previous studies,^15^, ^16^ to a 6‐month time horizon. This allowed us to examine the long‐term costs and consequences, such as those associated with the port implantation surgery, staff time for the first and following visits, patient monitoring, AEs, and other hospital resource usage.

We have also shown that HC is not just a cost‐saving health service; patients who received chemotherapy at home in fact have better health outcomes as measured by QALYs. This is owing to the fact that patients feel comfortable and secure at home because there is less disruption in their life. They have an increased feeling of control over the treatment and illness. Joo et al. has also shown that patients who were administered chemotherapy at home had higher satisfaction levels.^15^

Efficiency has always been a goal of the healthcare system. With an increasing number of patients being diagnosed with cancer, hospitals are more at risk of becoming overloaded if chemotherapy continues to be administered in the traditional way. Our study showed that the use of HC that started in 2016 at the Ramathibodi Hospital led to an increase in technical efficiency throughout the healthcare sector, as fewer inputs could produce high outputs. The benefits estimated from this study, however, do not extend to the fact that shifting some patients to HC could free some hospital beds for patients whose chemotherapy needs to be administered in a hospital. This additional benefit would have to be considered in future work. In this context, HC could also reduce the waiting time for treatment, thus, preventing delayed treatment, which can be expected to positively impact the outcome of cancer treatment and impede the progression of the disease.

The 2020 coronavirus disease (COVID‐19) pandemic has urged Thailand to implement infection prevention and control practices that include reduced exposure in hospitals.^17^ For cancer treatment, patients need to receive prolonged infusion chemotherapy at the hospital for at least 2–3 days, which could further increase the risk of COVID‐19 infection that already exists owing to their cancer.^18^ In light of this situation, in accordance with the results on HC's clinical effectiveness and economic evaluation revealed in this study, NHSO has supported HC treatment in Thailand and included it under their pilot program. Twenty‐three cancer hospitals have recently joined the pilot program (as of June, 2020), which supports HC reimbursement including costs for CV ports, port implantation surgery, ambulatory infusion pumps, and hospital management costs. All of these are crucial elements in the HC service.^18^

There are three limitations to this study. First, the analysis did not include the patients' out‐of‐pocket costs. Second, the AE results were retrieved from a single hospital. Finally, observations of costs and outcomes in this research were conducted at the Ramathibodi Hospital because it was the only hospital in Thailand with a long‐standing HC program operating since we began this study. Further, we conducted PSA with the AEs from the literature review; the results did not change and HC was further validated to be the dominant strategy. Nonetheless, it is possible that different results may have been obtained from other settings with other resource utilizations.

Even though the AEs in the HC and IP samples observed in our study were consistent with other studies, HC practice guidelines are important for successful implementation. The HC national practice guideline for reimbursement should include national standardization in chemotherapy regimens, as well as practice guidelines and protocols to prevent serious AEs.

## 5 CONCLUSION

In summary, conducting HC for a stage III colon cancer patient with CV port and portable infusion pump is an acceptable strategy due to its cost‐saving nature, from the societal perspective. The use of HC is sustainable in the context of Thailand, leading to a reduction in treatment costs and opportunity costs. HC is a good example of how the healthcare system could rethink, in an innovative way, to provide healthcare services more efficiently.

## CONFLICT OF INTEREST

The authors declare that there are no conflicts of interest in any form.

## ETHICAL ISSUE

The Institutional Review Board, Mahidol University, Faculty of Medicine, Ramathibodi Hospital approved the protocol (date of approval: 11/01/2018; no. MURA2018/867).

## Data Availability

The data that support the findings of this study are available on request from the corresponding author. The data are not publicly available due to privacy or ethical restrictions.
